# Continuously Monocropped Jerusalem Artichoke Changed Soil Bacterial Community Composition and Ammonia-Oxidizing and Denitrifying Bacteria Abundances

**DOI:** 10.3389/fmicb.2018.00705

**Published:** 2018-04-10

**Authors:** Xingang Zhou, Zhilin Wang, Huiting Jia, Li Li, Fengzhi Wu

**Affiliations:** ^1^Department of Horticulture, Northeast Agricultural University, Harbin, China; ^2^Key Laboratory of Biology and Genetic Improvement of Horticultural Crops, Northeast Region, Ministry of Agriculture, Harbin, China; ^3^Institute of Horticulture, Qinghai Academy of Agriculture and Forestry Sciences, Xining, China

**Keywords:** *Helianthus tuberosus* L., soil bacterial community, bacterial diversity, monocropping, nitrogen cycling

## Abstract

Soil microbial communities have profound effects on the growth, nutrition and health of plants in agroecosystems. Understanding soil microbial dynamics in cropping systems can assist in determining how agricultural practices influence soil processes mediated by microorganisms. In this study, soil bacterial communities were monitored in a continuously monocropped Jerusalem artichoke (JA) system, in which JA was successively monocropped for 3 years in a wheat field. Soil bacterial community compositions were estimated by amplicon sequencing of the 16S rRNA gene. Abundances of ammonia-oxidizing and denitrifying bacteria were estimated by quantitative PCR analysis of the *amoA*, *nirS*, and *nirK* genes. Results showed that 1–2 years of monocropping of JA did not significantly impact the microbial alpha diversity, and the third cropping of JA decreased the microbial alpha diversity (*P* < 0.05). Principal coordinates analysis and permutational multivariate analysis of variance analyses revealed that continuous monocropping of JA changed soil bacterial community structure and function profile (*P* < 0.001). At the phylum level, the wheat field was characterized with higher relative abundances of *Latescibacteria*, *Planctomycetes*, and *Cyanobacteria*, the first cropping of JA with *Actinobacteria*, the second cropping of JA with *Acidobacteria*, *Armatimonadetes*, *Gemmatimonadetes*, and *Proteobacteria*. At the genus level, the first cropping of JA was enriched with bacterial species with pathogen-antagonistic and/or plant growth promoting potentials, while members of genera that included potential denitrifiers increased in the second and third cropping of JA. The first cropping of JA had higher relative abundances of KO terms related to lignocellulose degradation and phosphorus cycling, the second cropping of JA had higher relative abundances of KO terms nitrous-oxide reductase and nitric-oxide reductase, and the third cropping of JA had higher relative abundances of KO terms nitrate reductase and nitrite reductase. The abundances of *amoA* genes decreased while *nirK* increased in the third cropping of JA, *nirS* continuously increased in the second and third cropping of JA (*P* < 0.05). Redundancy analysis and Mantel test found that soil organic carbon and Olsen phosphorus contents played important roles in shaping soil bacterial communities. Overall, our results revealed that continuous monocropping of JA changed soil bacterial community composition and its functional potentials.

## Introduction

The rapidly increasing global food demand poses a huge challenge for the sustainability of agricultural production (Tilman et al., 2011). Modern agricultural practices are often characterized by monocropping, which leads to the simplification of the components of agricultural systems (Cook, 2006). The continuous monocropping system, in which the same crop is repeatedly monocropped on the same land, is not long-term sustainable, because it usually results in reduction of crop yield and quality, a phenomenon which has been described as ‘soil sickness’ (Cook, 2006; van der Putten et al., 2013).

Plants can change soil biology, chemistry, and structure in ways that alter subsequent plant growth, and this process is referred as plant–soil feedback (van der Putten et al., 2013). As a kind of negative plant–soil feedback in agricultural ecosystems, soil sickness has been reported for several crops, such as corn (*Zea mays* L.) (Gentry et al., 2013), pea (*Pisum sativum* L.) (Nayyar et al., 2009) and cucumber (*Cucumis sativus* L.) (Zhou and Wu, 2012). Possible factors that contribute to soil sickness include accumulation of phytotoxic compounds, build-up of soil-borne pathogens, deterioration of soil physico-chemical characteristics, and changes in nutrient availability (Zhou and Wu, 2012; Huang et al., 2013). Recently, changes in soil biological properties have also been proposed to account for the yield decline in continuous monocropping systems (Nayyar et al., 2009; Huang et al., 2013; Zhou et al., 2017a).

Soil microorganisms are responsible for key processes associated with soil fertility and plant health, and are an important driver of the functioning of terrestrial ecosystems (Bever et al., 2012; Bhattacharyya and Jha, 2012). Changes in soil microbial communities may lead to alterations in the functions performed by the community and thus have feedbacks on plant health and fitness (Bever et al., 2012; Zhou et al., 2017a). Soil microbial communities are driven by a myriad of factors, including soil physical and chemical properties, aboveground plant species abundance and diversity, and agricultural practices (such as monocropping, crop rotation, intercropping, fertilization, irrigation, and tillage) (Acosta-Martínez et al., 2008; Nayyar et al., 2009; Rosenzweig et al., 2012; Sun et al., 2014; Zhou et al., 2017a). For example, it has been shown that diversified cropping systems (such as intercropping, crop rotation, and cover crop) usually have higher diversities and abundances of soil microbial communities than monocropping systems (Zhou et al., 2011, 2017a; Tiemann et al., 2015). Knowledge about how continuous monocropping influences soil microbial communities is helpful for the development of practices to relieve soil sickness in agriculture production.

Jerusalem artichoke (JA) (*Helianthus tuberosus* L.) is an economically important crop, which can be used as a food for direct human consumption and livestock feed after silage (Kaur and Gupta, 2002). Market forces have encouraged farmers to cultivate JA in monoculture and a reduction in tuber yield and quality was usually observed (Chi et al., 2009; Zhou et al., 2016). Previously, we found that continuous monocropping of JA changed the structure and decreased the diversity of soil bacterial communities as estimated by PCR-denaturing gradient gel electrophoresis analysis (Zhou et al., 2016). High-throughput sequencing techniques, such as 454 pyrosequencing and Illumina sequencing, can provide a higher resolution and a better understanding of environmental microbial communities than the PCR-based fingerprinting techniques (Schöler et al., 2017). High-throughput sequencing techniques also greatly facilitated the diversity and the composition analyses of microbial communities in agricultural soils (Acosta-Martínez et al., 2008; Sun et al., 2014; Bainard et al., 2016; Chavez-Romero et al., 2016; Zhou et al., 2017a). Therefore, it is necessary to deepen our understanding about the dynamic changes in soil microbial communities during continuous monocropping of crops with high-throughput sequencing techniques.

In this study, we evaluated the responses of soil bacterial communities to continuous monocropping of JA with high-throughput sequencing. JA was grown in a long-term cultivated wheat field for three successive years. Bulk soil bacterial communities were assessed by amplicon sequencing of the V3-V4 region of the 16S rRNA gene on an Illumina MiSeq platform. In addition, bacterial community potential functions were inferred from the amplicon data using Phylogenetic Investigation of Communities by Reconstruction of Unobserved States (PICRUSt) (Langille et al., 2013). Abundances of ammonia-oxidizing and denitrifying bacteria were estimated by quantitative PCR analysis of the *amoA*, *nirS*, and *nirK* genes. Plant litter and root exudates are important carbon resources for soil microorganisms, and the chemistries of these organic matters differ among plant species (Fierer et al., 2007; Eilers et al., 2010; Birouste et al., 2012). Therefore, we first hypothesized that soil bacterial communities differed between wheat- and JA-cultivated soils. Since it is usually observed that continuous monocropping had an adverse effect on soil bacterial community diversity, while crop rotation were able to increase soil bacterial community diversity (Zhou et al., 2011, 2017a; Tiemann et al., 2015). The first cropping of JA can be viewed as a wheat-JA rotation system. Therefore, our second hypothesis was that soils from the wheat field and the third cropping of JA would have lower soil bacterial community diversities than the first cropping of JA.

## Materials and Methods

### Field Experiment

The experimental site was located in field of Mojiaquanwan village, Chengbei District, Xining, China (36°42’N, 101°45’E), which has been continuously cultivated with wheat (*Triticum aestivum* L.) for more than 20 years. Wheat was grown from early March to early September and thereafter left fallow till next wheat crop. The soil was castanozem (FAO/UNESCO System of Soil Classification), containing organic matter: 2.03%, available N (NH_4_^+^ and NO_3_^-^): 69 mg kg^-1^, Olsen P: 65 mg kg^-1^, available K: 229 mg kg^-1^, EC (1:2.5, w/v) and pH (1:2.5, w/v), 8.12.

The field experiment was conducted from April 2010 to October 2012. The annual precipitations in these 3 years were 405.0, 390.4, and 446.1 mm, respectively, and the mean annual temperatures were 6.4, 5.7, and 5.2°C, respectively. There are four treatments in the experiment, namely, W, F, S, and T (**Table 1**). W was the long-term cultivated wheat field. F, S, and T were designed to be planted with JA for 1, 2, and 3 years, respectively. Briefly, in 2010, treatment T was planted with JA, the other three treatments were planted with wheat. In 2011, treatments T and S were planted with JA, the other two treatments were planted with wheat. In 2012, treatments T, S, and F were planted with JA, the treatment W was planted with wheat. The experiment was set up in a randomized block design, with three replicate plots for each treatment. Each plot measured 120 m long and 80 m wide.

**Table 1:** Experiment setup of the field experiment.

Treatments	Year		
	
	2010	2012	2013
W	Wheat	Wheat	Wheat
F	Wheat	Wheat	JA
S	Wheat	JA	JA
T	JA	JA	JA


*Crops, wheat or Jerusalem artichoke (JA), were planted in different treatments from 2010 to 2012. W was continuously planted with wheat; F, S, and T were planted with JA for 1, 2, and 3 years, respectively. All soil samples were taken in 2013.*

Jerusalem artichoke tubers (cv. Qingyu 2), provided by Institute of Horticulture, Qinghai Academy of Agriculture and Forestry Sciences, China, were planted on April 5 each year and harvested on October 25 each year. Within-row spacing was 40 cm and the row width was 60 cm. Wheat was broadcast seeded in early March and harvested in early September. There was one crop (wheat or JA) per year. After the harvest of JA and wheat, the fields were left fallow until to plant the next crop. Both diammonium hydrogen phosphate and urea were applied at the rate of 300 kg ha^-1^ as basal fertilizer. Flooding irrigation with groundwater was performed when necessary. Weeds were manually removed once a month in May and June.

### Soil Sampling and DNA Extraction

Bulk soil samples were collected on November 25, 2012, 1 month after JA harvest. Eight soil cores (5 cm diameter, 15 cm deep) were randomly collected between rows of crops from each plot to make a composite sample. Large stones and root debris were removed by sieving (2 mm), then fresh soils were transported to laboratory and stored at -70°C. There were triplicate soil samples for each treatment and there were 12 soil samples in total.

Total soil DNA was extracted with the PowerSoil DNA Isolation Kit (MO BIO Laboratories, Carlsbad, CA, United States) as per the manufacturer’s instructions. Each composite soil sample was extracted in triplicate and the extracted DNA solutions were pooled.

### High-Throughput Amplicon Sequencing and Data Processing

Soil bacterial community compositions were analyzed with high-throughput sequencing on an Illumina MiSeq platform. Primers of F338/R806 were used to amplify V3-V4 region of the bacterial 16S rRNA gene as described before (Derakhshani et al., 2016; Zhou et al., 2017a). Both the forward and reverse primers also had a 6-bp barcode unique to each sample, which were used to permit multiplexing of samples. Each composite soil sample was independently amplified in triplicate, the products of the triplicate PCR reactions were pooled and purified using the Agarose Gel DNA purification kit (TaKaRa, China). Then, purified amplicons were quantified by a TBS-380 micro fluorometer with Picogreen reagent (Invitrogen, United States), and pooled in equal amounts. The mixture was then paired-end sequenced (2 × 300) on an Illumina Miseq platform at Majorbio Bio-Pharm Technology Co., Ltd., Shanghai, China.

Raw sequence reads were de-multiplexed, quality-filtered, and processed using FLASH (Magoc and Salzberg, 2011) as described before (Zhou et al., 2017a). Operational taxonomic units (OTUs) were delineated at 97% sequence similarity with USEARCH using an agglomerative clustering algorithm (Edgar, 2010). Then, a representative sequence of each OTU was taxonomically classified through BLAST against the SILVA (Quast et al., 2013). Chimeric sequences were identified and removed using USEARCH 6.1 in QIIME 1.9.1 (Caporaso et al., 2010). Functions of soil bacterial communities were predicted by PICRUSt from the 16S rRNA marker gene sequences on the Galaxy platform^1^ (Langille et al., 2013), and the biological functions were annotated in the KEGG database (Kanehisa et al., 2012). Specifically, we focused on functions associated with carbon, nitrogen, phosphorus, and sulfur cycling. The data set was deposited in the NCBI-Sequence Read Archive with the submission Accession Number SRP115368.

### Quantitative PCR Analysis

Abundances of ammonia-oxidizing and denitrifier communities were estimated by quantitative PCR assays with an IQ5 real-time PCR system (Bio-Rad Lab, Los Angeles, CA, United States). For the ammonia-oxidizing community, the gene encoding ammonia monooxygenase catalytic subunit A (*amoA*) was amplified using the primer set of *amoA*1F/*amoA*2R (Rotthauwe et al., 1997) according to the methods described by Glaser et al. (2010). For the denitrifier community, the cytochrome *cd*1-containing nitrite reductase gene (*nirS*) and the Cu-dependent nitrite reductase gene (*nirK*) were amplified using the primer sets of *nirS*Cd3aF/*nirS*R3cd (Kandeler et al., 2006) and *nirK*1F/*nirK*5R (Braker et al., 1998), respectively, according to the methods described before (Hai et al., 2009; Braker et al., 2015). A 20 μl PCR reaction mixture contained 10 μl of 2× Real SYBR Mixture (Tiangen Biotech, Beijing, China), 0.2 μM of each primer, 2.5 ng of soil DNA. Standard curves were created with 10-fold dilution series of plasmids containing the ITS regions from soil samples. The specificity of the products was confirmed by melting curve analysis and agarose gel electrophoresis. The threshold cycle (Ct) values obtained for each sample were compared with the standard curve to determine the initial copy number of the target gene. Sterile water was used as a negative control to replace the template. All amplifications were performed in triplicate.

### Statistical Analysis

Read counts from high-throughput amplicon sequencing were not rarefied to equal sampling depths because this unnecessarily discards data (McMurdie and Holmes, 2014). For alpha diversity analysis, square root transformed read counts (Balint et al., 2015) were used to calculated Hill’s series of diversity. The series consists of three numbers: N0 is the number of species in a sample; N1 is the antilogarithm of the Shannon diversity (representing the abundant species in a sample); and N2 is the inverse Simpson diversity (representing the very abundant species in a sample) (Hill, 1973). To compare with alpha diversity indices from the unrarefied data, alpha diversity indices were also calculated from a randomly selected subset of 22,503 16S rRNA gene sequences per sample.

For beta diversity analysis, read counts were centered log-ratio (CLR) transformed (Fernandes et al., 2014). Bacterial community structure and function profile were analyzed using principal coordinates analysis (PCoA) based on a Euclidean distance matrix. Permutational multivariate analysis of variance (PerMANOVA) was used to test the differences in microbial communities with the Euclidean distance and 999 permutations. The PCoA and PerMANOVA analyses were performed with the pcoa and adonis functions in ‘vegan’ package in ‘R’ (Version 3.3.1), respectively.

Linear discriminant effect size (LEfSe) analysis was used to identify biomarkers that were significantly associated with each treatment with an alpha value of 0.05 for the Kruskal–Wallis test and a threshold of 2.0 for logarithmic linear discriminant analysis (LDA) scores (Segata et al., 2011).

Previously, we found that continuously monocropped JA did not change soil pH and inorganic N content, the first cropping of JA had the highest soil organic carbon (SOC) content while the third cropping of JA had the lowest soil Olsen P (Zhou et al., 2017b). Redundancy analysis (RDA) was used to identify soil properties that predict the variation of bacterial communities. Mantel test with a Monte Carlo simulation with 999 randomizations was used to assess the relationships between the Euclidean distance of bacterial community and soil chemical properties. RDA and Mantel test analyses were performed with the rda function in the ‘vegan’ package and the mantel.rtest function in the ‘ade4’ package in ‘R’ (version 2.1.3), respectively. Spearman’s rank correlations between soil properties and relative abundances of bacterial classes and genus, and predicted functions were calculated in ‘psych’ package in ‘R’ (Version 3.3.1).

Differences in Hill’s series of diversity, relative abundances of microbial taxa and abundances of *amoA*, *nirS*, and *nirK* genes among treatments were analyzed using one-way analysis of variance (ANOVA) followed by Tukey’s honestly significant difference (HSD) test at the 0.05 probability level.

## Results

### Amplicon Sequencing Data

After filtering reads by basal quality control and removing singletons, Illumina Miseq sequencing of bacterial 16S rRNA gene fragments generated 322,976 quality bacterial sequences with an average read length of 397 bp, and 22,503–30,340 sequences were obtained per sample (mean = 26,915). The Good’s coverage, which reflects the captured diversity, was larger than 98% for all samples (data not shown). Rarefaction curves of OTUs at 97% sequence similarity and Shannon’s diversity indices of all samples tended to approach the saturation plateau (Supplementary Figure S1), which indicates that the majority of the bacterial diversity was recovered by the surveying effort.

### Bacterial Community Composition

**FIGURE 1:**
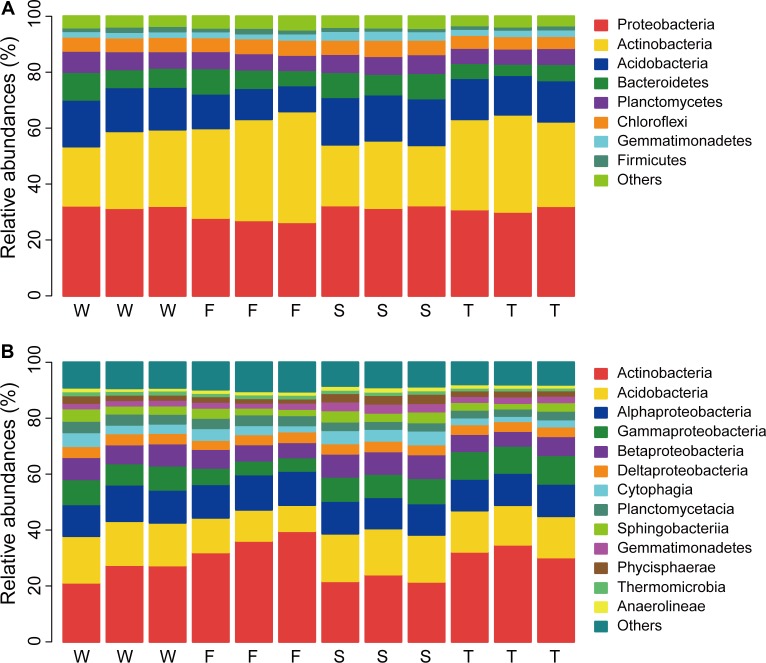
Relative abundances of main soil bacterial phyla **(A)** and classes **(B)**. W represents the wheat field; F, S, and T represent the first, second, and third cropping of Jerusalem artichoke (JA), respectively.

In total, 32 phyla were detected across all samples and 0.99% bacterial sequences were unclassified at the phylum level (Unclassified Bacteria). The dominant phyla (relative abundance > 5%) across all soil samples were *Proteobacteria*, *Actinobacteria*, *Acidobacteria*, *Bacteroidetes*, *Planctomycetes*, and *Chloroflexi*, which accounted for more than 92% of the bacterial sequences (**Figure 1A**). The top three phyla were *Proteobacteria*, *Actinobacteria*, and *Acidobacteria*, which had relative abundances ranging from 26.36 to 32.37%, 21.17 to 39.64%, and 9.31 to 17.00%, respectively. *Gemmatimonadetes* and *Firmicutes* were less abundant phyla (relative abundance < 5% but > 1%) with relative abundances ranging from 1.76 to 3.25% and 1.13 to 2.00%, respectively. Groups of *Nitrospirae*, *Verrucomicrobia*, *Cyanobacteria*, *Latescibacteria*, *Armatimonadetes*, and *JL-ETNP-Z39* were also detected at relatively low abundances in all samples (relative abundance > 0.1%).

**FIGURE 2:**
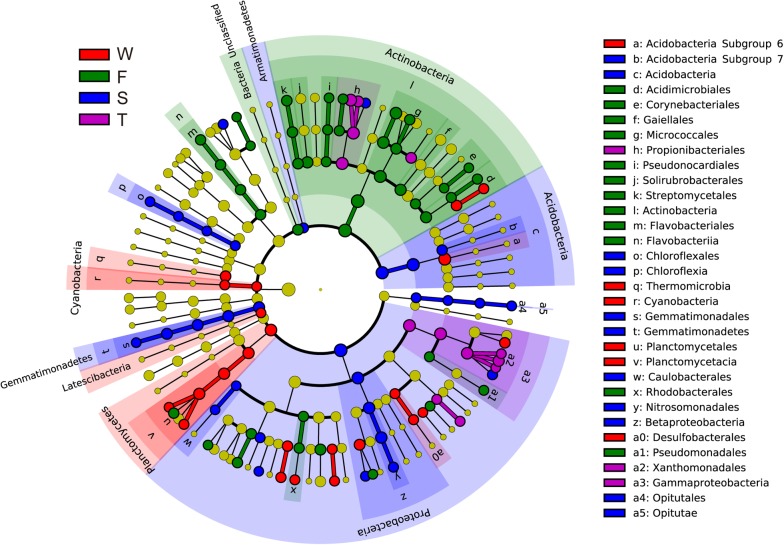
Cladograms, generated from LEfSe analysis, represent the polygenetic distribution of soil bacterial taxa. Biomarkers that are significantly associated with each treatment with LDA scores larger than 2 are shown. Significantly discriminant taxon nodes are colored: red for the wheat field (W), green, blue and purple for the first (F), second (S), and third (T) cropping of Jerusalem artichoke (JA), respectively. Yellow circles represent non-significant differences in abundance between treatment groups for that particular taxon. Each circle’s diameter is proportional to the taxon’s abundance. Labels are shown of the phylum, class and order levels. The LDA scores of each identified biomarker from the phylum to genus levels are shown in Supplementary Figure S1.

Linear discriminant effect size analysis identified 107 differentially abundant taxa from the phylum to the genus level (**Figure 2** and Supplementary Figure S2). The first cropping of JA had the most (41) and the third cropping of JA has the least (13) number of differentially abundant taxa. Among these differentially abundant taxa, nine were found to be genus-level biomarkers. At the phylum level, the wheat field was enriched with *Latescibacteria*, *Planctomycetes*, and *Cyanobacteria*; the first cropping of JA with *Actinobacteria* and Unclassified Bacteria; the second cropping of JA with *Acidobacteria*, *Armatimonadetes*, *Gemmatimonadetes*, and *Proteobacteria* (*P* < 0.05).

At the class level, more than 70 bacterial taxa were detected. All samples were dominated by *Actinobacteria*, *Acidobacteria*, *Alphaproteobacteria*, *Gammaproteobacteria*, and *Betaproteobacteria* (**Figure 1B**). LEfSe analysis identified 11 biomarkers at the class level (**Figure 2** and Supplementary Figure S2). The wheat field was characterized with higher relative abundances of *Thermomicrobia*, *Cyanobacteria*, and *Planctomycetacia*; the first cropping of JA with *Actinobacteria* and *Flavobacteriia*; the second cropping of JA with *Acidobacteria*, *Chloroflexia*, *Gemmatimonadetes*, *Betaproteobacteria*, and *Opitutae*; the third cropping of JA with *Gammaproteobacteria* (*P* < 0.05).

At the genus level, more than 500 bacterial taxa were detected. *Arthrobacter*, *Blastococcus*, *Chryseolinea*, *Gaiella*, *Lysobacter*, *Marmoricola*, *Nocardioides*, *Skermanella*, and *Streptomyces* spp. were dominant classified genera (relative abundance > 1%) (**Table 2**). The relative abundances of *Variibacter*, *Illumatobacter*, *Altererythrobacter*, and *Gemmata* spp. were higher in the wheat field than in other treatments (*P* < 0.05) (**Table 2** and Supplementary Figure S2). The relative abundances of *Microbacterium*, *Mycobacterium*, *Pseudonocardia*, *Algoriphagus*, *Flavobacterium*, *Bosea*, *Microvirga*, and *Pseudomonas* spp. were higher in the first cropping of JA than in other treatments (*P* < 0.05). The relative abundances of *Adhaeribacter*, *Roseiflexus*, *Gemmatimonas*, *Rhizobium*, *Caenimonas*, and *Nitrosospira* spp. were higher in the second cropping of JA than in other treatments (*P* < 0.05). The relative abundances of *Nitrospira*, *Marmoricola*, *Nocardioides*, *Haliangium*, and *Lysobacter* were higher in the third cropping of JA than in other treatments (*P* < 0.05).

**Table 2:** The relative abundances of main classified bacterial genera (average relative abundance > 0.3%) in the wheat field (W), the first (F), second (S), and third (T) cropping of Jerusalem artichoke (JA).

	W	F	S	T		W	F	S	T
*Nocardioides*	4.73 ± 0.48 ^b^	5.53 ± 0.47 ^b^	3.99 ± 0.13 ^b^	8.32 ± 0.42 ^a^	*Ferruginibacter*	0.66 ± 0.13 ^a^	0.53 ± 0.09 ^a^	0.57 ± 0.05 ^a^	0.58 ± 0.09 ^a^
*Arthrobacter*	1.53 ± 0.08 ^b^	6.77 ± 0.29 ^a^	2.10 ± 0.09 ^b^	6.20 ± 0.37 ^a^	*Blastocatella*	0.57 ± 0.12 ^ab^	0.32 ± 0.03 ^b^	0.89 ± 0.07 ^a^	0.51 ± 0.05 ^b^
*Skermanella*	1.89 ± 0.07 ^a^	2.20 ± 0.15 ^a^	1.89 ± 0.07 ^a^	2.00 ± 0.05 ^a^	*Iamia*	0.67 ± 0.06 ^ab^	0.69 ± 0.04 ^a^	0.41 ± 0.06 ^c^	0.46 ± 0.01 ^bc^
*Gaiella*	2.18 ± 0.19 ^ab^	2.36 ± 0.16 ^a^	1.75 ± 0.13 ^ab^	1.61 ± 0.05 ^b^	*Arenimonas*	0.63 ± 0.04 ^a^	0.48 ± 0.03 ^b^	0.71 ± 0.02 ^a^	0.37 ± 0.01 ^b^
*Lysobacter*	1.46 ± 0.03 ^b^	0.57 ± 0.00 ^c^	1.67 ± 0.05 ^b^	3.28 ± 0.21 ^a^	*Planctomyces*	0.56 ± 0.05 ^a^	0.50 ± 0.04 ^ab^	0.45 ± 0.02 ^ab^	0.35 ± 0.03 ^b^
*Marmoricola*	1.23 ± 0.11 ^bc^	1.72 ± 0.18 ^b^	0.94 ± 0.08 ^c^	2.34 ± 0.15 ^a^	*Illumatobacter*	0.69 ± 0.03 ^a^	0.52 ± 0.03 ^b^	0.33 ± 0.01 ^c^	0.29 ± 0.01 ^c^
*Chryseolinea*	1.85 ± 0.30 ^a^	0.97 ± 0.15 ^b^	1.71 ± 0.07 ^ab^	1.41 ± 0.15 ^ab^	*Microlunatus*	0.50 ± 0.06 ^ab^	0.62 ± 0.07 ^a^	0.31 ± 0.03 ^b^	0.33 ± 0.02 ^b^
*Blastococcus*	1.43 ± 0.21 ^a^	1.83 ± 0.19 ^a^	1.20 ± 0.04 ^a^	1.38 ± 0.10 ^a^	*Flavobacterium*	0.48 ± 0.03 ^b^	0.97 ± 0.07 ^a^	0.17 ± 0.01 ^c^	0.12 ± 0.00 ^c^
*Streptomyces*	0.89 ± 0.06 ^b^	1.44 ± 0.07 ^a^	1.42 ± 0.09 ^a^	0.77 ± 0.04 ^b^	*Haliangium*	0.42 ± 0.01 ^b^	0.29 ± 0.04 ^b^	0.43 ± 0.05 ^ab^	0.57 ± 0.02 ^a^
*Nitrospira*	0.92 ± 0.06 ^b^	0.89 ± 0.06 ^b^	0.95 ± 0.03 ^b^	1.23 ± 0.05 ^a^	*Bryobacter*	0.41 ± 0.04 ^a^	0.34 ± 0.06 ^a^	0.52 ± 0.04 ^a^	0.40 ± 0.03 ^a^
*Acidibacter*	1.19 ± 0.13 ^a^	0.60 ± 0.04 ^b^	0.92 ± 0.07 ^ab^	0.75 ± 0.10 ^b^	*Aeromicrobium*	0.35 ± 0.02 ^b^	0.23 ± 0.02 ^c^	0.48 ± 0.02 ^a^	0.48 ± 0.03 ^a^
*Pir4 lineage*	0.87 ± 0.04 ^a^	0.94 ± 0.03 ^a^	0.67 ± 0.03 ^b^	0.84 ± 0.02 ^a^	*Caenimonas*	0.35 ± 0.04 ^bc^	0.42 ± 0.02 ^b^	0.53 ± 0.02 ^a^	0.17 ± 0.02 ^c^
*Microvirga*	0.97 ± 0.09 ^a^	1.02 ± 0.07 ^a^	0.63 ± 0.01 ^b^	0.63 ± 0.06 ^b^	*Nitrosospira*	0.35 ± 0.03 ^b^	0.38 ± 0.04 ^b^	0.58 ± 0.05 ^a^	0.13 ± 0.03 ^c^
*Agromyces*	0.59 ± 0.06 ^a^	0.72 ± 0.07 ^a^	0.67 ± 0.02 ^a^	1.07 ± 0.03 ^a^	*Amaricoccus*	0.48 ± 0.07 ^a^	0.43 ± 0.04 ^a^	0.19 ± 0.01 ^b^	0.33 ± 0.01 ^ab^
*Microbacterium*	0.92 ± 0.08 ^b^	1.02 ± 0.06 ^a^	0.52 ± 0.04 ^c^	0.49 ± 0.08 ^c^	*Adhaeribacter*	0.35 ± 0.01 ^b^	0.32 ± 0.02 ^b^	0.50 ± 0.03 ^a^	0.15 ± 0.01 ^c^
*Variibacter*	0.86 ± 0.03 ^a^	0.66 ± 0.01 ^b^	0.61 ± 0.02 ^b^	0.64 ± 0.04 ^b^	*Pedomicrobium*	0.35 ± 0.01 ^a^	0.29 ± 0.01 ^a^	0.32 ± 0.03 ^a^	0.36 ± 0.02 ^a^
*Pirellula*	0.79 ± 0.06 ^a^	0.70 ± 0.04 ^ab^	0.68 ± 0.03 ^ab^	0.55 ± 0.04 ^b^	*Opitutus*	0.28 ± 0.02 ^bc^	0.36 ± 0.03 ^ab^	0.43 ± 0.01 ^a^	0.21 ± 0.01 ^c^
*Steroidobacter*	0.62 ± 0.06 ^ab^	0.42 ± 0.04 ^b^	0.78 ± 0.07 ^a^	0.66 ± 0.11 ^ab^	*Altererythrobacter*	0.53 ± 0.05 ^a^	0.16 ± 0.01 ^c^	0.34 ± 0.02 ^b^	0.21 ± 0.02 ^bc^
*Solirubrobacter*	0.63 ± 0.09 ^ab^	0.87 ± 0.12 ^a^	0.41 ± 0.04 ^b^	0.56 ± 0.04 ^ab^	*Bacillus*	0.34 ± 0.04 ^a^	0.35 ± 0.03 ^a^	0.21 ± 0.04 ^a^	0.33 ± 0.01 ^a^
*Bradyrhizobium*	0.56 ± 0.03 ^a^	0.58 ± 0.05 ^a^	0.64 ± 0.03 ^a^	0.66 ± 0.01 ^a^	*Nordella*	0.20 ± 0.01 ^b^	0.19 ± 0.00 ^b^	0.39 ± 0.02 ^a^	0.43 ± 0.05 ^a^


*Values (mean ± SE, *n* = 3) with different letters are significantly different at the 0.05 probability level.*

A total of 2,395 OTUs were identified at 97% similarity. Most dominated OTUs, with relative abundances greater than 0.5% of the total sequences, were mainly assigned to the *Acidobacteria*, *Actinobacteria*, and *Gammaproteobacteria* at the class level (Supplementary Table S1). The relative abundances of one OTUs assigned to *Acidibacter*, *Chryseolinea*, *Lysobacter*, *Acidobacteria Subgroup 6 norank*, uncultured *Nitrosomonadaceae* and unclassified *Xanthomonadaceae* were higher in the wheat field than in the first cropping of JA; while the relative abundances of OTUs assigned to *Arthrobacter* and *Streptomyces* spp. was higher in the first cropping of JA than in the wheat field (*P* < 0.05). The third cropping of JA had the highest relative abundances of two OTUs assigned to *Nocardioides*, *Marmoricola* and *Lysobacter* spp. and the lowest *Comamonadaceae* unclassified among all treatments (*P* < 0.05).

### Bacterial Community Diversity and Structure

**FIGURE 3:**
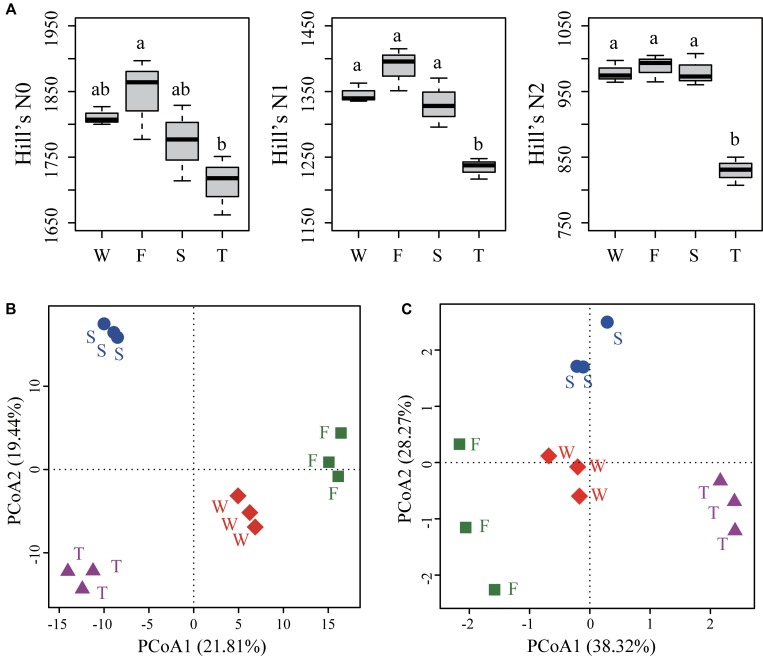
Alpha diversities **(A)** and PCoA analyses of soil bacterial community structure **(B)** and function profile **(C)**. **(A)** Hill’s series of diversity was calculated from unrarefied data. Different letters indicate significant difference based on Tukey’s HSD test (*P* < 0.05). **(B)** The PCoA analysis of soil bacterial community structure was based on the Euclidean distance of the centered log-ratio (CLR) transformed read counts at the OTU level. **(C)** The PCoA analysis of soil bacterial community function profile was based on the Euclidean distance of the CLR transformed pathway numbers at KEGG level 3. OTUs were delineated at 97% sequence similarity. W represents the wheat field; F, S, and T represent the first, second, and third cropping of Jerusalem artichoke (JA), respectively.

For alpha diversities calculated from unrarefied data (**Figure 3A**) and rarefied data (Supplementary Figure S3), the number of OTUs (Hill’s N0) was lower in the third cropping of JA than in the first cropping of JA (ANOVA, *P* < 0.05). Hill’s N1 and N2 were significantly lower in third cropping of JA than in other treatments (ANOVA, *P* < 0.05).

The PCoA analysis at the OTU level showed a clear separation among samples from the wheat field, the first, second, and third cropping of JA (**Figure 3B**). PerMANOVA analysis demonstrated that continuous cropping of JA significantly changed soil bacterial community structure (*F* = 3.034, *R*^2^ = 0.532, *P* < 0.001).

### Predicted Functions of Bacterial Communities

**FIGURE 4:**
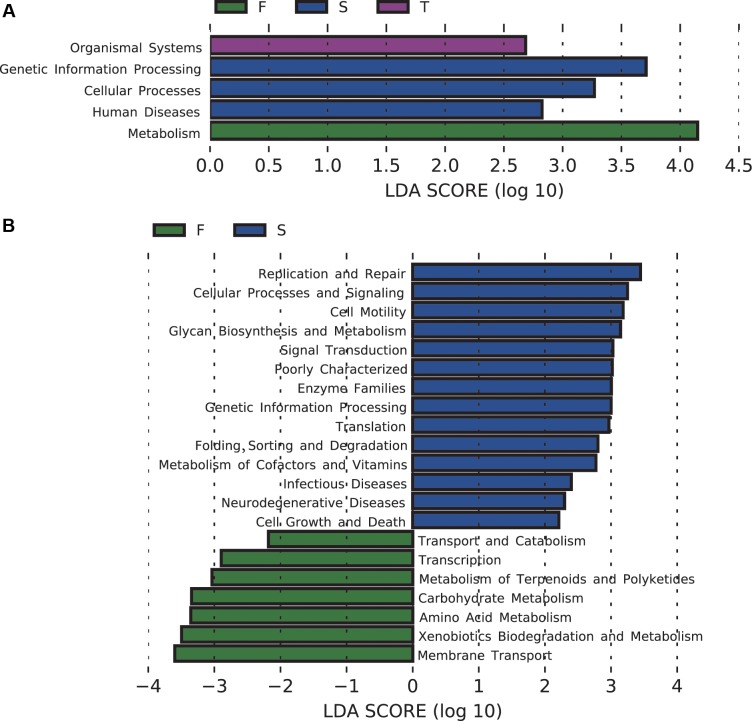
Changes in soil bacterial community functional profiles. Histograms **(A,B)** showed the LDA scores calculated for the differentially abundant biomarkers at KEGG level 1 and 2, respectively (*P* < 0.05). W represents the wheat field; F, S, and T represent the first, second, and third cropping of Jerusalem artichoke (JA), respectively.

The majority of the predicted functional gene categories were related to metabolism (52.09%), followed by genetic information (15.76%), environmental information processing (13.27%), and unclassified (12.91%). LEfSe analysis identified five differentially abundant KEGG pathways at KEGG level 1, 21 differentially abundant KEGG pathways at KEGG level 2 (**Figure 4**), and 89 differentially abundant KEGG pathways at KEGG level 3 (Supplementary Figure S4). The first cropping of JA was characterized by enrichment of functions related to metabolism at KEGG level 1, and amino acid metabolism and carbohydrate metabolism at KEGG level 2; the second cropping of JA was characterized by enrichment of functions related to genetic information processing and cellular processes at KEGG level 1; the third cropping of JA was characterized by enrichment of functions related to organismal systems at KEGG level 1 (**Figure 4**).

For pathways at level 3 that are involved in carbohydrate metabolism, pentose and glucuronate interconversions, galactose metabolism, inositol phosphate metabolism, glyoxylate and dicarboxylate metabolism, fructose and mannose metabolism, glycolysis/gluconeogenesis, pyruvate metabolism, propanoate metabolism, butanoate metabolism, were enriched in the first cropping of JA; TCA cycle was enriched in the third cropping of JA. At KEGG level 3, the second cropping of JA was also enriched with sulfur metabolism.

**FIGURE 5:**
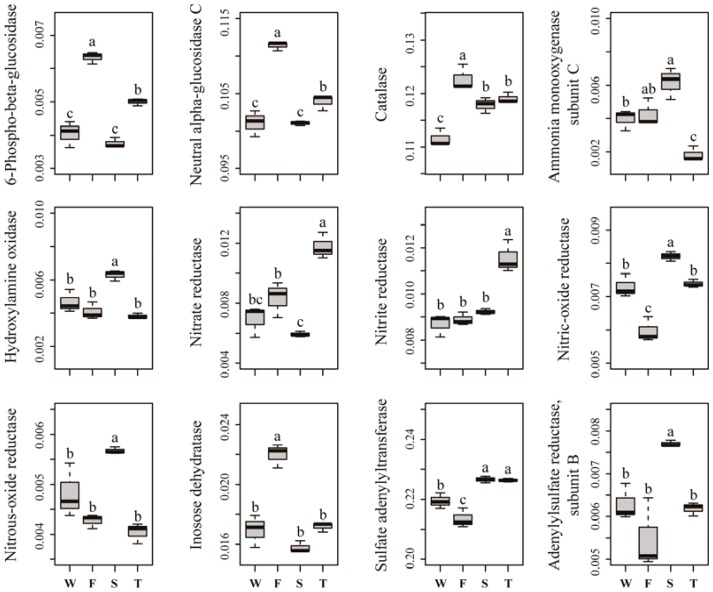
Relative abundances of significantly changed predicted KEGG Orthologs related to carbon, nitrogen, phosphorus, and sulfur cycling. Different letters indicate significant difference based on Kruskal–Wallis test (*P* < 0.05). W represents the wheat field; F, S, and T represent the first, second, and third cropping of Jerusalem artichoke (JA), respectively.

Several predicted KEGG Ortholog (KO) terms related to carbon, nitrogen, phosphorus and sulfur cycling were also differed among treatments (**Figure 5**). For example, the relative abundances of KO terms related to lignocellulose degradation, such as 6-phospho-beta-glucosidase, neutral alpha-glucosidase C and catalase, were higher in the first cropping of JA than in other treatments (*P* < 0.05). The relative abundances of KO terms ammonia monooxygenase subunit C and hydroxylamine oxidase, which are involved in ammonification, were higher in the second cropping of JA than in the wheat field and third cropping of JA (*P* < 0.05). Among all treatments, the third cropping of JA had the highest relative abundances of KO terms nitrate reductase and nitrite reductase, while the second cropping of JA had the highest relative abundances of KO terms nitrous-oxide reductase and nitric-oxide reductase (*P* < 0.05). The relative abundance of KO term inosose dehydratase, which participates in phosphorus cycling was higher in the first cropping of JA than in other treatments (*P* < 0.05). For KO terms related to sulfur cycling, the relative abundance of sulfate adenylyltransferase and adenylylsulfate reductase subunit B were higher in the second cropping of JA than in the wheat field and the first cropping of JA (*P* < 0.05).

Principal coordinates analysis showed that soil bacterial function profiles at KEGG level 3 differed among samples from the wheat field, the first, second, and third cropping of JA (**Figure 3C**). PerMANOVA analysis also demonstrated that continuous cropping of JA significantly changed the function of soil bacterial communities (*F* = 6.478, *R*^2^ = 0.708, *P* < 0.001).

### Relationships Between Soil Bacterial Communities and Soil Chemical Properties

**FIGURE 6:**
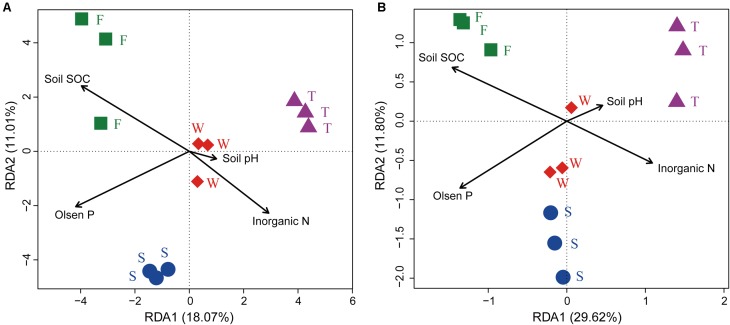
Ordination plots of the results from the redundancy analysis. **(A)** Relationship between soil bacterial community structures at the OTU level (97% sequence similarity) with soil chemical properties. **(B)** Relationship between soil bacterial function profiles at KEGG level 3 with soil chemical properties. W represents the wheat field; F, S, and T represent the first, second, and third cropping of Jerusalem artichoke (JA), respectively.

The RDA analysis and Mantel test were conducted to identify the key drivers of soil bacterial community structure and function profile. In the RDA plots of both bacterial community structure and function, soil SOC, inorganic N, and Olsen P had longer arrows than the soil pH (**Figure 6**). Mantel test demonstrated that soil bacterial community structure was significantly correlated to SOC (*r* = 0.339, *P* = 0.002) and Olsen P (*r* = 0.395, *P* = 0.004) but not to inorganic N (*r* = 0.395, *P* = 0.105) and soil pH (*r* = 0.075, *P* = 0.238); soil bacterial function profile was significantly correlated to SOC (*r* = 0.406, *P* = 0.003) and Olsen P (*r* = 0.426, *P* = 0.004) but not to inorganic N (*r* = 0.126, *P* = 0.220) and soil pH (*r* = -0.085, *P* = 0.692). Spearman’s rank correlation test showed that the relative abundances of bacterial class Gammaproteobacteria was negatively correlated with SOC (*r* = -0.85, *P* < 0.05), and genus *Caenimonas* spp. was positively correlated with SOC (*r* = 0.82, *P* < 0.05) and Olsen P (*r* = 0.83, *P* < 0.05). The relative abundance of the relative abundances of KO terms neutral alpha-glucosidase C and inosose dehydratase were positively correlated with SOC (*r* = 0.75, *P* < 0.05; *r* = 0.75, *P* < 0.05, respectively). The relative abundance of KO term ammonia monooxygenase subunit C was positively correlated with Olsen P (*r* = 0.71, *P* < 0.05).

### Soil Ammonia-Oxidizing and Denitrifier Community Abundances

**FIGURE 7:**
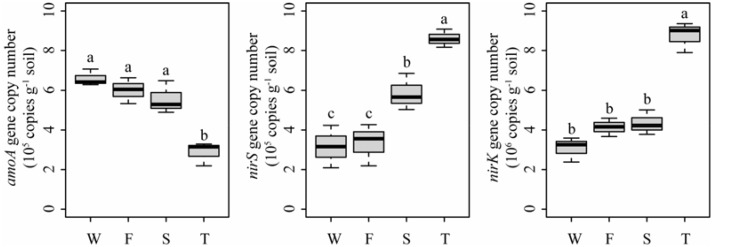
Abundances of *amoA*, *nirS*, and *nirK* genes in the wheat field (W), the first (F), second (S), and third (T) cropping of Jerusalem artichoke (JA) as determined by quantitative PCR. Different letters indicate significant difference based on Tukey’s HSD test (*P* < 0.05).

Quantitative PCR analysis showed that the ammonia-oxidizing abundance, expressed as *amoA* gene copy number, was significantly lower in the third cropping of JA than in other treatments (*P* < 0.05) (**Figure 7**). However, the denitrifier community abundance, expressed as *nirS* and *nirK* gene copy numbers, was significantly higher in the third cropping of JA than in other treatments (*P* < 0.05). Meanwhile, *nirS* gene copy number was higher in the second cropping of JA than in the wheat field and the first cropping of JA (*P* < 0.05).

## Discussion

The productivity and sustainability of agricultural system depend greatly on the functional processes carried out by soil microorganisms (Bever et al., 2012). Mounting evidences demonstrated that agricultural practices, such as fertilization, tillage, crop rotation, and intercropping, could alter soil microbial community (Garbeva et al., 2008; Zhou et al., 2011, 2017a; Bever et al., 2012). The present study stressed the influences of continuous monocropping on soil bacterial communities by amplicon sequencing of the 16S rRNA marker gene. PCoA and PerMANOVA analyses revealed that soil bacterial community composition and function profile changed during continuous monocropping of JA, which supported our first hypothesis.

It has been observed that soil microbial community can become compositionally adapted to utilize certain plant litter type (Ayres et al., 2009). Some bacterial taxa [such as *Gemmata* spp. (Bastian et al., 2009)], that were involved in degrading wheat residues, were enriched in the wheat field. These results indicate that long-term wheat cultivation selected for specific microorganisms that can degrade wheat residues. Several other bacterial taxa that associated with decomposing plant-derived organic matters were also enriched in other treatments. For example, *Bosea* and *Pseudonocardia* spp. were enriched in the first cropping of JA; *Adhaeribacter* and *Gemmatimonas* spp. were enriched in the second cropping of JA; *Lysobacter* and *Nocardioides* spp. were enriched in the third cropping of JA. Previous studies demonstrated that *Adhaeribacter* (Bastian et al., 2009), *Gemmatimonas* (Bastian et al., 2009), *Pseudonocardia* (Espana et al., 2011), *Lysobacter* (Chavez-Romero et al., 2016) were involved in decomposition of crop residues, such as potato (*Solanum tuberosum* L.), rice, maize and wheat; Species in *Bosea* (Houfani et al., 2017) have cellulolytic activities. Moreover, continuous monocropping of JA altered the predicted bacterial functions related to lignocellulose degradation. This would be possibly explained by the species-specific effects of plants on soil microbial communities through varying quantities and qualities of plant-derived organic matters, which can be used as substrates by soil microorganisms, as the chemistries of plant-derived organic matters were shown to differ among plant species (Fierer et al., 2007; Meier and Bowman, 2008; Eilers et al., 2010; Bever et al., 2012; Birouste et al., 2012).

Linear discriminant effect size analysis revealed that the first cropping of JA were enriched with bacterial species with pathogen-antagonistic and/or plant growth promoting potentials, including *Bosea* (Cavalca et al., 2010), *Microbacterium* (Bhattacharyya and Jha, 2012), *Mycobacterium* (Hayat et al., 2010), and *Pseudonocardia* spp. (Nimnoi et al., 2010). In our experiment, soils from the wheat field mainly contained wheat debris while soils from the JA-cultivated fields contained crop debris from both wheat and JA. It was found that most wheat residues (more that 80%) was decomposed within 320 days after wheat residues incorporated into the soil (Cookson et al., 1998). Thus, the diversity of plant-derived organic matters may be higher in the first cropping of JA, which also had higher soil SOC (Zhou et al., 2017b). Therefore, our results were in line with previous studies showing that increasing resource quantity and quality through increasing the temporal and spatial plant diversity can enhance the function of soil microbial communities (Rosenzweig et al., 2012; Tiemann et al., 2015). In our experiment, the first cropping of JA can be viewed as a wheat-JA rotation system. Thus, wheat-JA rotation may be used in production to stimulate soil bacteria beneficial to plants.

Quantitative PCR showed that the third cropping of JA has the lowest *amoA* gene copy number but had the highest *nirS* and *nirK* gene copy number. The second cropping of JA had higher *nirS* gene copy number than the wheat field and the first cropping of JA. This may be attributed to the lower available P in the third cropping of JA since soil P availability play an important in modulating soil N cycle. For example, it has been reported that nitrification was dependent on P availability (Sierra et al., 2003) and poor P availability can promote denitrification at higher N fertilizer inputs (Baral et al., 2014). PICRUSt revealed that continuous monocropping of JA altered the predicted bacterial functions related to nitrogen cycling. Specifically, the relative abundances of one KO term related to ammonification (ammonia monooxygenase subunit C) was lower in the third cropping of JA than in other treatments. Meanwhile, the relative abundances of KO terms of nitrate reductase and nitrite reductase were higher in the third cropping of JA. The relative abundances of KO terms of nitrous-oxide reductase and nitric oxide reductase were higher in the second cropping of JA. *Caenimonas*, *Gemmatimonas*, and *Rhodopirellula* spp. were enriched in the second cropping of JA. *Haliangium*, *Marmoricola*, and *Nocardioides* spp. were enriched in the third cropping of JA. Members of these taxa were reported to be involved in denitrification. For example, *Gemmatimonas* (Coyotzi et al., 2016) and *Rhodopirellula* spp. (Coyotzi et al., 2016) were shown to possess nitrite reductase gene and nitrous-oxide reductase gene, while *Arenimonas* spp. (Remmas et al., 2016) harbors nitrite reductase gene. Denitrifying strains have been described in *Caenimonas* (Ryu et al., 2008), *Haliangium* (McIlroy et al., 2016), *Marmoricola* (Dastager et al., 2008), and *Nocardioides* spp. (Woo et al., 2012). These indicated that soil nitrogen cycling may be changed by continuously monocropped JA.

Several studies have reported that soil edaphic properties, especially soil pH, were important determinants of soil bacterial community structures (Fierer and Jackson, 2006; Liu et al., 2014). However, the present study found that soil bacterial community structure was not correlated to soil pH. This may be due to the fact that soil pH was relatively stable in our cropping system (Zhou et al., 2017b). The first cropping of JA had higher soil SOC (Zhou et al., 2017b) and was characterized with higher relative abundance of *Actinobacteria* and lower relative abundance of *Acidobacteria*, which was in agreement with others’ finding that Actinobacteria responded positively while *Acidobacteria* responded negatively to exogenously applied labile carbon resources (Fierer et al., 2007; Eilers et al., 2010). The third cropping of JA had lower soil Olsen P (Zhou et al., 2017b) and higher relative abundance of *Marmoricola* spp., which was consistent with previous studies reporting that these bacterial taxa had negative relationship with soil P (Sun et al., 2014; Bainard et al., 2016). Our RDA analysis and Mantel test also confirmed that soil SOC played an important role in shaping soil bacterial communities, which was in accordance with the observation that soil carbon and P status are important factors in structuring soil bacterial communities (Griffiths et al., 2011; Liu et al., 2014).

Generally, it is suggested that intensive agricultural practices, such as continuous monocropping, had adverse effects on soil microbial community diversity (Zhou et al., 2011, 2017a; Tiemann et al., 2015; Tsiafouli et al., 2015). Our results showed that the third cropping of JA had lower bacterial community diversity indices than the first cropping of JA. However, the wheat field and the first cropping of JA had similar bacterial community diversity indices. Therefore, our second hypothesis was only partially validated. Accumulating evidence suggests that increasing soil microbial diversity can have positive effects on pathogen suppression, nutrient cycling, and plant growth (Bever et al., 2012). Therefore, the declined bacterial community diversity in the third cropping of JA maybe associated with the soil sickness in JA production. Long-term monocropping of several crops can induce soil suppressiveness against soil-borne diseases (Berendsen et al., 2012). For example, the decline of take-all of wheat, caused by *Gaeumannomyces graminis* var. *tritici*, has been observed during wheat monocropping (de Souza et al., 2003). The induction of soil suppressiveness was associated with the build-up of antagonistic microorganisms, such as fluorescent *Pseudomonas* spp. and increased bacterial community diversity (de Souza et al., 2003; Rosenzweig et al., 2012). However, it was not known whether suppressive soil was induced in our wheat field and its relationship with soil bacterial diversities, which should be stressed in future studies.

One shortcoming of this experiment was that soil samples in only one time point were analyzed. Environmental variables, which change across seasons, are main governors of soil microbial communities (Bell et al., 2009). It has been demonstrated that there were seasonal variations in the effects of agricultural practices on soil microbial communities (Spedding et al., 2004; Wolsing and Prieme, 2004). Therefore, seasonal changes in soil bacterial communities in our continuously monocropped JA system should be investigated in more detail. Agricultural weeds were shown to affect soil microbial functional group abundance and community composition (Wortman et al., 2013). However, weeds were only manually removed in the early growth season of JA and the total amount of weeds on the field was not measured in this study. Therefore, there was possibility that the total of weeds differed among treatments and contributed to the changes in soil bacterial communities observed in our cropping system.

## Conclusion

In summary, our results demonstrated that continuous monocropping of JA changed soil bacterial community composition and function profile, and soil bacterial community diversity was lower the third cropping of JA. Soil SOC and Olsen P were the important predictors of soil bacterial community in our cropping system. Our results also suggested that wheat rotated with JA can stimulate potentially beneficial bacteria. Soil microbial community composition and function are tightly linked (Bever et al., 2012). However, we only predicted bacteria function from a taxonomy assignment in this study (Langille et al., 2013). Further researches should focus on getting direct evidence of changes in soil microbial functions in our continuously monocropped JA system through approaches such as metagenomic or metatranscriptomic sequencing (Choi et al., 2017; Ofaim et al., 2017).

## Author Contributions

XZ, LL, and FW conceived and designed the study. XZ, ZW, and HJ performed the experiments. XZ analyzed the data and wrote the manuscript. All authors read and approved the final manuscript.

## Conflict of Interest Statement

The authors declare that the research was conducted in the absence of any commercial or financial relationships that could be construed as a potential conflict of interest.

## Footnotes

1 http://huttenhower.sph.harvard.edu/galaxy
