# Supplementary material for: Continuously Monocropped Jerusalem Artichoke Changed Soil Bacterial Community Composition and Ammonia-Oxidizing and Denitrifying Bacteria Abundances
Source: Front Microbiol. 2018 Apr 10;9:705. doi: 10.3389/fmicb.2018.00705 (PMC5902710; doi:10.3389/fmicb.2018.00705)
Supplement: Supplementary file 1 [file Data_Sheet_1.DOC]

**Continuously monocropped Jerusalem artichoke changed soil bacterial community composition and ammonia-oxidizing and denitrifying bacteria abundances**

Xingang Zhou^1,2,3^, Zhilin Wang^1^, Huiting Jia^1^, Li Li^3^, Fengzhi Wu^1,2^

^1^Department of Horticulture, Northeast Agricultural University, Harbin, China

^2^Key Laboratory of Biology and Genetic Improvement of Horticultural Crops (Northeast Region), Ministry of Agriculture, Harbin, China

^3^Institute of Horticulture, Qinghai Academy of Agriculture and Forestry Sciences, Xining, China

Correspondence and requests for materials should be addressed to F.W. (fzwuneau@yahoo.com).

**Figure legends**

**FIGURE S1** Rarefaction analysis of soil bacterial communities in the continuously monocropped Jerusalem artichoke system. (a) Rarefaction curves of OTUs at 97% sequence similarity. (b) Rarefaction curves of Shannon’ diversity indices. W represents the wheat field; F, S and T represent the first, second and third cropping of Jerusalem artichoke, respectively.

**FIGURE S2** The LDA scores of each identified biomarker from the phylum to genus levels in each treatment from the LEfSe analysis of soil bacterial community composition (P<0.05, LDA>2.0). Detected taxa with relative abundance >0.3% in at least one sample were used as inputs for LEfSe analysis. W represents the wheat field; F, S and T represent the first, second and third cropping of Jerusalem artichoke, respectively.

**FIGURE S3** Alpha diversity of bacterial communities based on rarefied data. Hill’s series of diversity was calculated from a randomly selected subset of 22,503 16S rRNA gene sequences per sample. Different letters indicate significant difference based on Tukey’s HSD test (P<0.05).

**FIGURE S4** The LDA scores of each identified biomarker at KEGG level 3 in each treatment from the LEfSe analysis of soil bacterial function profile (P<0.05, LDA>2.0). Functional pathways were inferred from OTUs using PICRUSt and annotated with KEGG database. W represents the wheat field; F, S and T represent the first, second and third cropping of Jerusalem artichoke, respectively.

**FIGURE S1**

**
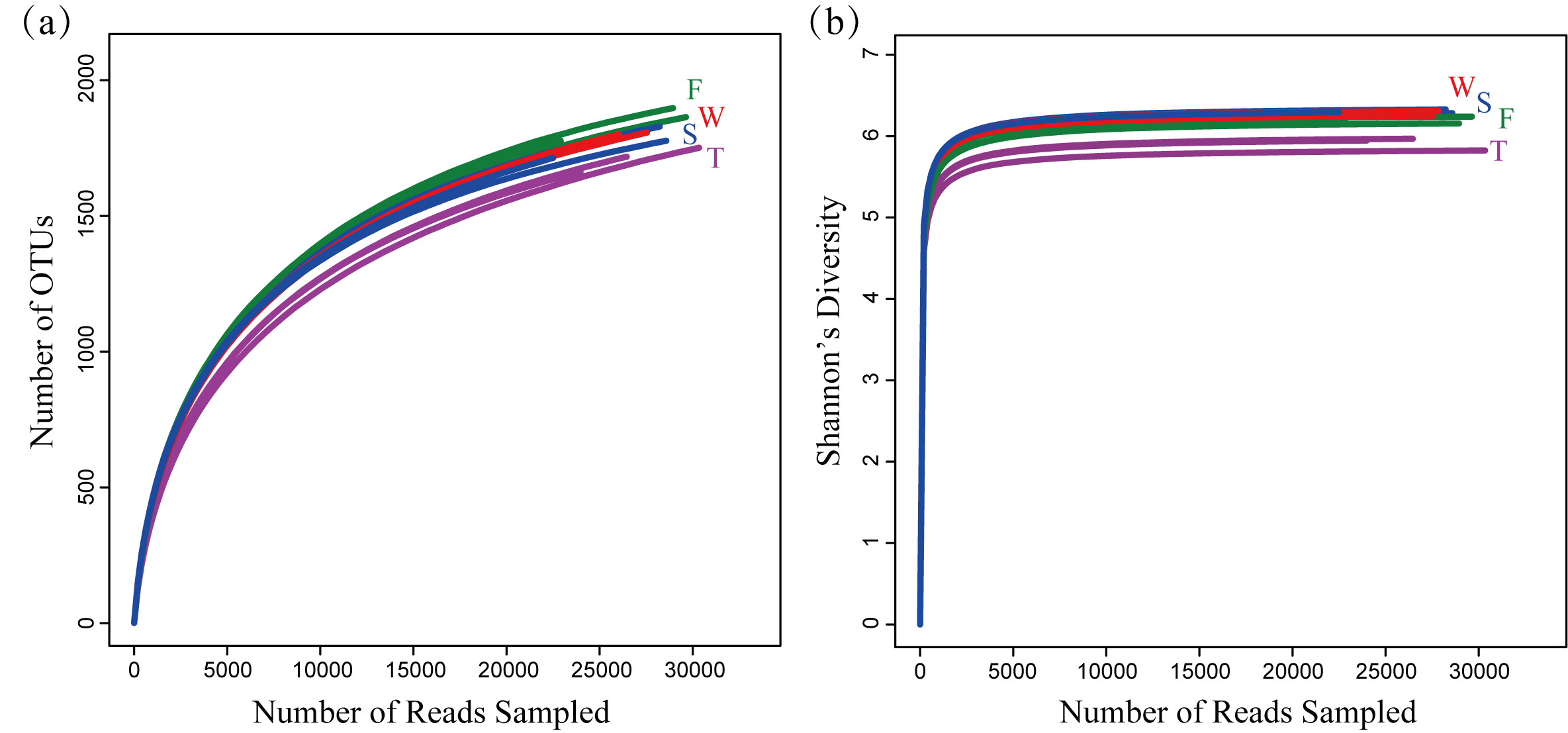
**

**FIGURE S2**


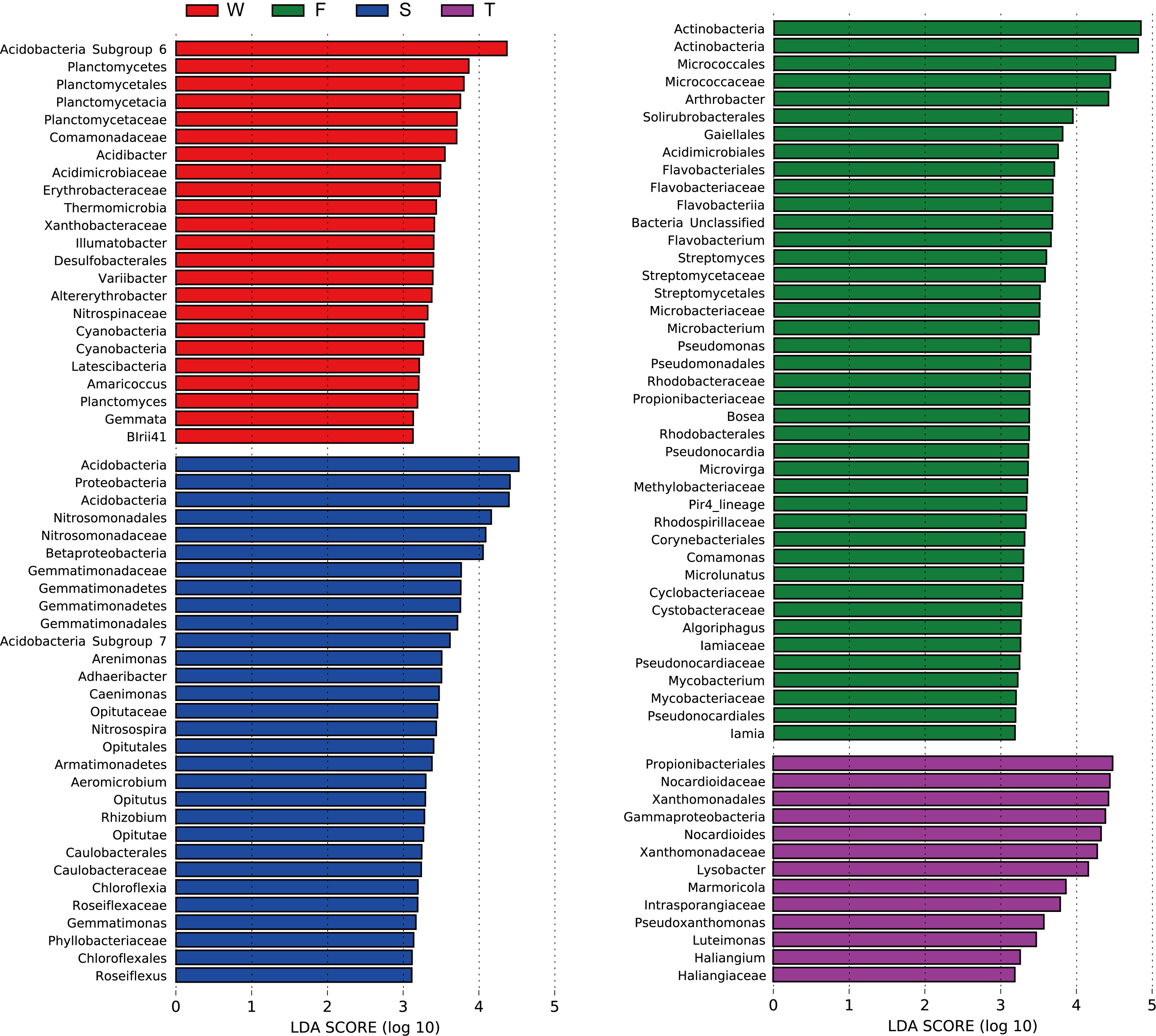


**FIGURE S3**


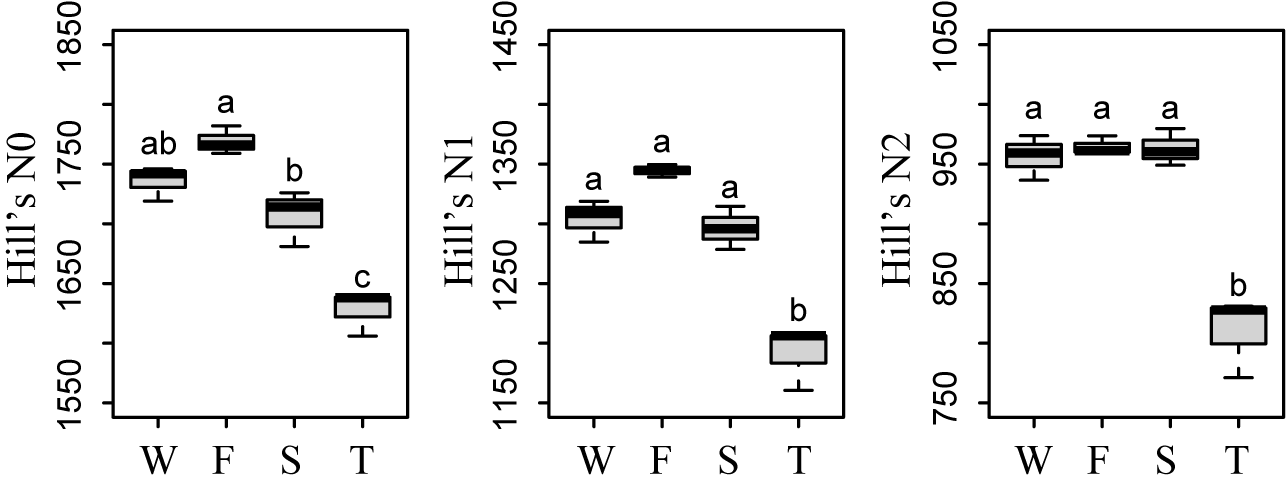


**FIGURE S4**


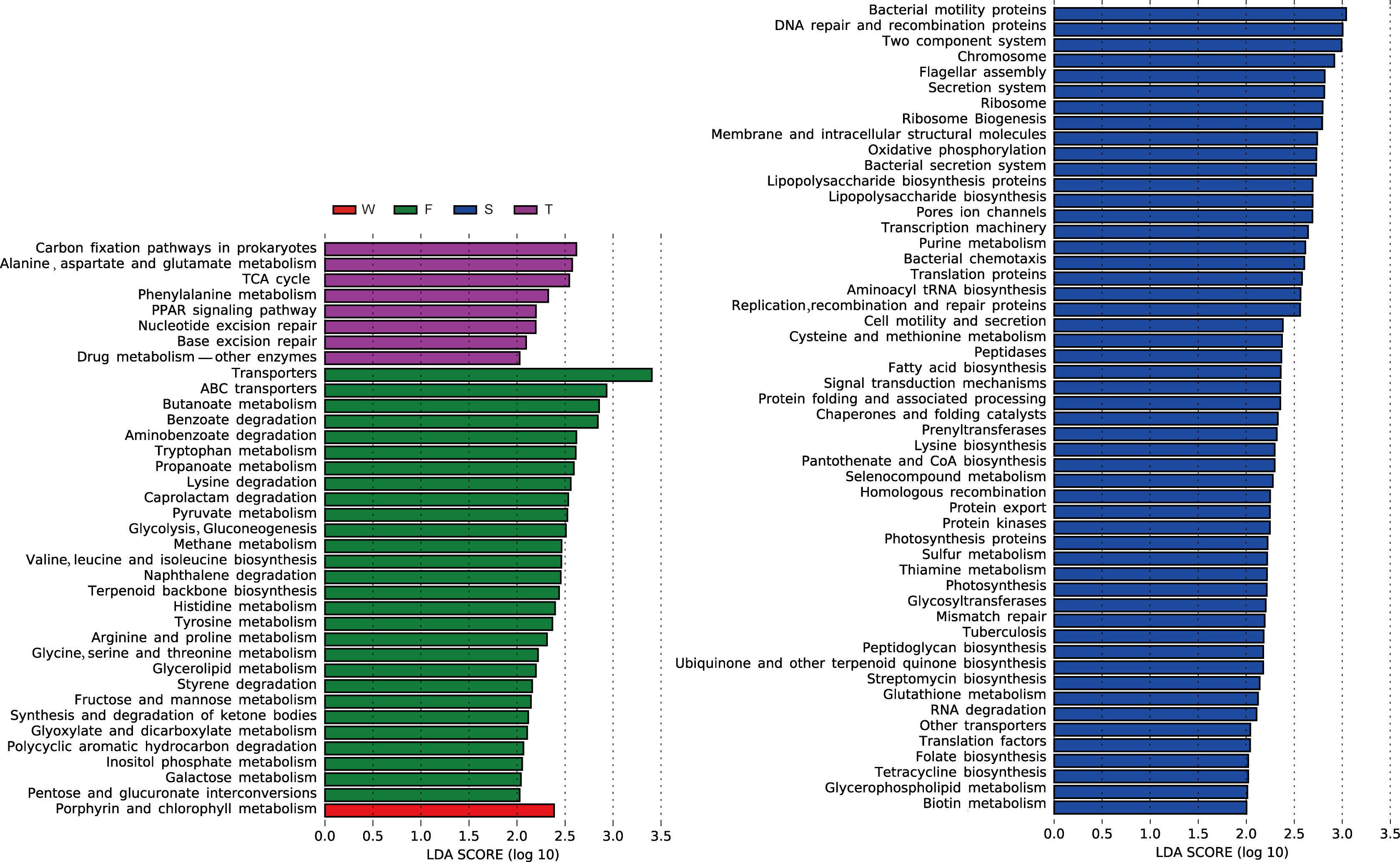


**Table S1** The most abundant soil bacterial OTUs in the wheat field (W), the first (F), second (S) and third (T) cropping of Jerusalem artichoke. OTUs were delineated at 97% sequence similarity. Only OTUs with relative abundances greater than 0.5% of the total sequences were reported. Values (mean±SE, n=3) with different letters are significantly different (P<0.05).

| Phylum | Class | Genus/Species | Relative abundances (%) | | | |
| --- | --- | --- | --- | --- | --- | --- |
|  |  |  | W | F | S | T |
| *Actinobacteria* | *Actinobacteria* | *Arthrobacter* | 1.52±0.08 b | 6.73±0.29 a | 2.09±0.09 b | 6.19±0.37 a |
| *Actinobacteria* | *Actinobacteria* | *Nocardioides* | 1.48±0.14 b | 1.70±0.19 b | 1.36±0.09 b | 2.54±0.08 a |
| *Actinobacteria* | *Actinobacteria* | *Nocardioides* | 1.35±0.16 b | 1.49±0.12 b | 1.11±0.01 b | 2.72±0.09 a |
| *Actinobacteria* | *Actinobacteria* | *Marmoricola* | 1.23±0.11 bc | 1.72±0.18 b | 0.94±0.08 c | 2.34±0.15 a |
| *Acidobacteria* | *Acidobacteria* | Subgroup 6 norank | 1.50±0.07 b | 0.97±0.09 c | 1.67±0.03 ab | 1.92±0.04 a |
| *Acidobacteria* | *Acidobacteria* | Subgroup 6 norank | 1.83±0.16 a | 1.28±0.13 b | 1.79±0.06 a | 0.99±0.01 b |
| *Actinobacteria* | *Actinobacteria* | *Blastococcus* | 1.43±0.21 a | 1.83±0.19 a | 1.20±0.04 a | 1.38±0.10 a |
| *Actinobacteria* | *Actinobacteria* | *Nocardioides* | 1.40±0.12 ab | 1.67±0.16 a | 0.95±0.04 b | 1.72±0.14 a |
| *Bacteroidetes* | *Cytophagia* | *Chryseolinea* | 1.33±0.21 a | 0.70±0.11 b | 1.40±0.06 a | 1.01±0.10 ab |
| *Proteobacteria* | *Betaproteobacteria* | uncultured *Nitrosomonadaceae* | 1.17±0.05 a | 0.45±0.01 b | 1.22±0.08 a | 1.33±0.05 a |
| *Proteobacteria* | *Gammaproteobacteria* | unclassified *Xanthomonadaceae* | 0.85±0.09 b | 0.35±0.05 c | 1.27±0.08 a | 1.56±0.09 a |
| *Proteobacteria* | *Alphaproteobacteria* | *Skermanella* | 1.00±0.04 a | 1.14±0.08 a | 0.92±0.04 a | 0.97±0.06 a |
| *Actinobacteria* | *Actinobacteria* | *Streptomyces* | 0.63±0.04 b | 1.05±0.06 a | 1.03±0.09 a | 0.50±0.03 b |
| *Proteobacteria* | *Gammaproteobacteria* | *Acidibacter* | 1.10±0.13 a | 0.55±0.03 b | 0.83±0.06 ab | 0.66±0.08 b |
| *Actinobacteria* | *Actinobacteria* | *Microbacterium* | 0.92±0.08 a | 1.02±0.06 a | 0.52±0.04 b | 0.49±0.08 b |
| *Actinobacteria* | *Actinobacteria* | uncultured *Intrasporangiaceae* | 0.82±0.07 a | 0.94±0.12 a | 0.30±0.02 b | 0.78±0.06 a |
| *Acidobacteria* | *Acidobacteria* | Subgroup 6 norank | 0.85±0.06 a | 0.45±0.03 c | 0.58±0.02 bc | 0.72±0.06 ab |
| *Proteobacteria* | *Gammaproteobacteria* | *Lysobacter* | 0.56±0.03 b | 0.22±0.01 c | 0.54±0.04 b | 1.28±0.09 a |
| *Proteobacteria* | *Gammaproteobacteria* | uncultured *Xanthomonadales* | 0.82±0.11 a | 0.39±0.03 b | 0.54±0.02 ab | 0.81±0.10 a |
| *Proteobacteria* | *Alphaproteobacteria* | *Bradyrhizobium* | 0.56±0.03 a | 0.58±0.05 a | 0.64±0.03 a | 0.66±0.01 a |
| *Acidobacteria* | *Acidobacteria* | Subgroup 6 norank | 0.58±0.02 b | 0.48±0.06 bc | 0.86±0.01 a | 0.40±0.02 c |
| *Proteobacteria* | *Alphaproteobacteria* | *Skermanella* | 0.48±0.02 a | 0.61±0.04 a | 0.58±0.02 a | 0.62±0.03 a |
| *Chloroflexi* | *Thermomicrobia* | uncultured *Thermomicrobia* | 0.59±0.03 a | 0.54±0.03 ab | 0.56±0.04 ab | 0.46±0.02 b |
| *Proteobacteria* | *Gammaproteobacteria* | *Steroidobacter* | 0.55±0.05 a | 0.38±0.03 a | 0.64±0.06 a | 0.57±0.09 a |
| *Acidobacteria* | *Acidobacteria* | Subgroup 6 norank | 0.60±0.03 a | 0.38±0.05 b | 0.55±0.02 a | 0.57±0.02 a |
| *Proteobacteria* | *Gammaproteobacteria* | uncultured *Xanthomonadales* | 0.77±0.09 a | 0.31±0.02 b | 0.49±0.06 ab | 0.51±0.07 ab |
| *Proteobacteria* | *Betaproteobacteria* | unclassified *Comamonadaceae* | 0.71±0.03 a | 0.53±0.04 b | 0.48±0.01 b | 0.30±0.05 c |
